# Toxicity of a Botanical Mixture Based on *Salvia guaranitica* and *Capsicum annuum* Extracts Against the Leafhopper *Scaphoideus titanus* Ball, 1932

**DOI:** 10.3390/insects17050520

**Published:** 2026-05-20

**Authors:** Domenico Rongai, Maria Gabriella Di Serio

**Affiliations:** CREA Research Centre for Engineering and Agro-Food Processing, via Nazionale 38, 65012 Cepagatti, Italy; mariagabriella.diserio@crea.gov.it

**Keywords:** natural compounds, insecticidal activity, *flavescence dorée*, vegetal oil, *Salvia guaranitica*, *Capsicum annuum*

## Abstract

The American grapevine leafhopper (*Scaphoideus titanus*) is a major threat to grapevine production, as it is the primary vector of the *flavescence dorée* phytoplasma. Its ongoing spread poses a serious risk to several viticultural regions, including Italy. This study evaluates the efficacy of a plant-derived formulation composed of extracts of *Salvia guaranitica*, *Capsicum annuum* ‘Cayenne’, and olive oil (Form) against *S. titanus*, through laboratory bioassays and field trials conducted in 2024 and 2025. In laboratory assays, Form applied at 0.5% caused 84.0% mortality in *S. titanus* adults, while at 2% mortality reached 94.3%. In field trials, prior to treatment (1 August 2024), the average number of adults captured was 3.9 per trap in both vineyard blocks. Four days after treatment (12 August 2024), captures decreased significantly to two per trap in the treated vineyard, whereas they increased to 8.2 per trap in the untreated block. Eight days after treatment (16 August 2024), captures in the treated vineyard remained low (2.4 per trap), while the untreated vineyard maintained high levels (eight per trap). Nymph monitoring showed a similar trend. Before treatment, the incidence of *S. titanus* was 44.0% and 46.0% in untreated and Form-treated plants, respectively. After treatment (8 June 2025), the percentage of leaves hosting nymphs rose to 84.0% in the untreated vineyard but remained stable at 44.0% in the Form-treated area. Overall, these results demonstrate that Form exerts a measurable toxic effect on *S. titanus* under both laboratory and field conditions. The findings suggest that a plant-derived formulation such as Form represents a promising alternative to chemical insecticides for controlling the main vector of *flavescence dorée* and could be integrated into sustainable management strategies.

## Simple Summary

The American grapevine leafhopper (*Scaphoideus titanus*), a vector of *flavescence dorée*, threatens European viticulture. A plant-derived formulation containing *Salvia guaranitica*, *Capsicum annuum*, and olive oil (Form) was tested in laboratory and field trials. Form caused 84.0–94.3% adult mortality in laboratory assays and significantly reduced adult captures and nymph incidence in treated vineyards. These results indicate that Form provides effective control of *S. titanus* and represents a promising plant-based alternative to chemical insecticides for sustainable vineyard management.

## 1. Introduction

The American grapevine leafhopper (*Scaphoideus titanus*) represents a threat to grapevine production, being the main vector of the grapevine *flavescence dorée* phytoplasma [1,2,3]. Its continuous expansion makes this leafhopper a serious threat to several countries, and mandatory insecticide treatments against this pest have been introduced in Europe. *S. titanus* overwinters as an egg embedded in the rhytidome of wood that is two or more years old [4]. Hatching occurs, staggered throughout the late spring–early summer, generally starting in mid-May, with variations depending on the environment and the growing year. Before becoming an adult, the insect develops through five nymphal instars. The juvenile stages preferentially inhabit moist, shaded microhabitats and are predominantly located on the abaxial surface of leaves borne by suckers along the grapevine trunk, whereas adults are distributed throughout the vineyard canopy [5]. The insect after suckering, following heavy rains, can be found on the wild herbaceous plant layer. *S. titanus* is very sensitive to insecticide treatments: it only takes two treatments with phosphate esters over the course of a summer to bring populations to very low levels regardless of the formulation [6]. Traditional insecticides, which were highly effective against *S. titanus* in the past, can no longer be used due to restrictions imposed by EU authorities. Chemical control must be supplemented with cultivation techniques that reduce the population, such as removing branches from winter pruning and suckers [7,8]. In Italian viticulture, only a limited number of active substances are authorized for the control of *S. titanus*. The control strategy varies according to the region and the production system (conventional or organic), and includes pyrethroids, neonicotinoids, potassium salts of fatty acids, *Beauveria bassiana*, and sweet orange oil. Pyrethrins, despite their low persistence and the consequent need for repeated applications, are widely used [9,10], and their efficacy may be enhanced when applied in combination with suitable adjuvants.

Among conventional insecticides, the most effective compounds reported to date include deltamethrin, lambda-cyhalothrin, sulfoxaflor, and etofenprox, although their residual activity appears to be limited. Field trials have identified etofenprox and deltamethrin as the most effective options for managing adult populations of *S. titanus* [8].

In organically managed vineyards, satisfactory control levels with natural pyrethrins combined with piperonyl butoxide can be achieved only when certain application guidelines are strictly followed, such as performing treatments during evening hours and applying high water volumes to ensure optimal canopy coverage [11].

A rational control strategy can be implemented through early monitoring of *S. titanus* populations in order to identify the optimal timing for insecticide applications. Abandoned vineyards and American vines can be a source of vectors for vineyards in the vicinity and should be removed before the adults appear as they can move up to 300 m into neighboring vineyards [12]. Innovative techniques are needed to reduce insecticide use and improve control effectiveness. *Capsicum annuum* berries, which are rich in alkaloids, flavonoids, anthocyanins, tannins, and saponins, exhibit a high level of insecticidal activity [13]. This activity has been largely attributed to the presence of terpenoids such as eugenol and pulegone [14]. Eugenol, in particular, has been shown to be toxic to a range of insect pests, including the corn weevil (*Sitophilus zeamais*), the red flour beetle (*Tribolium castaneum*), the larger grain borer (*Prostephanus truncatus*), and the American cockroach (*Periplaneta americana*) [15].

Olive oil is also known to exert harmful effects on insects, primarily through the obstruction of spiracles, which interferes with respiration [16]. However, as reported in previous studies, the plant extracts included in Form exhibit only limited insecticidal activity when applied individually under field conditions [17,18,19,20,21,22]. Alternative sustainable methods or strategies could support the control of *S. titanus*, in line with the European Union’s requirements for the sustainable use of pesticides. The need for innovative and environmentally compatible tools is particularly relevant in the context of *flavescenza dorée*, one of the most destructive phytoplasma-associated diseases in European viticulture. The spread of *flavescenza dorée* depends almost entirely on the presence and abundance of its main vector, *S. titanus*, whose feeding activity allows the acquisition and transmission of the phytoplasma from infected vines to healthy plants.

In recent years, the progressive reduction in authorized insecticidal active ingredients and the stricter restrictions introduced by European legislation have increased the difficulty of maintaining vector populations below epidemiologically critical thresholds. Moreover, the management of *flavescenza dorée* requires a combination of measures including compulsory removal of infected vines, surveillance activities, and targeted vector control making the search for sustainable alternatives to conventional insecticides even more urgent. In this scenario, natural or plant-derived products that can reduce vector abundance or interfere with vector behavior may represent valuable tools to complement existing control programs, particularly in integrated and organic production systems. Such approaches could contribute not only to reducing chemical input but also to slowing the spread of *flavescenza dorée* by limiting vector movement, survival, or feeding activity.

In this study, we investigated the efficacy of a natural product for the control of *S. titanus* by conducting a series of targeted bioassays under controlled laboratory conditions together with complementary field trials. Attention was given to evaluating the performance of a botanical formulation designed as an alternative to conventional insecticides currently used in European vineyards for the management of the American grapevine leafhopper, the primary vector of *flavescence dorée*. The formulation tested in this work consists of extracts of *Salvia guaranitica* and *C. annuum*, combined with olive oil to enhance solubility, stability, and adherence to the plant surface.

This combined mixture was selected based on previous evidence indicating the insecticidal or repellent properties of individual plant components, and on the hypothesis that their joint application may result in enhanced biological activity. Laboratory bioassays were conducted to assess direct toxicity at different concentrations, while field evaluations were carried out to test the efficacy of the formulation under realistic vineyard conditions. The field trials were implemented during the 2024 and 2025 growing seasons, allowing us to examine the formulation’s performance against different developmental stages of adults in 2024 and nymphs in 2025 and across varying environmental parameters. Together, these complementary approaches provided a comprehensive assessment of the formulation’s potential as a botanical tool for integrated pest management in viticulture.

## 2. Materials and Methods

### 2.1. Insect Collection and Rearing

Adults of *S. titanus* were collected using a sweep net in a vineyard located in Chieti (Italy) in July of the years 2024–2025. The insects were reared on a small grapevine plant placed inside a net cage (L 75 cm × W 75 cm × H 115 cm; mesh size 44 µm × 34 µm) until laboratory experiments were conducted. The rearing cage was maintained in a greenhouse without active control of temperature or humidity to simulate natural field conditions; however, temperatures typically ranged between 24 and 28 °C and relative humidity between 60 and 70%, values generally considered suitable for the survival and normal activity of *S. titanus*.

### 2.2. Preparation of Formulation (Form)

The formulation consisted of an emulsion containing 10% Hot Pepper-Infused Oil (HPIO) dispersed in gum arabic, added to a 0.6% aqueous solution of *S. guaranitica* extract (SGE). Arabic gum acted as a natural emulsifier, ensuring the stable dispersion of the oil phase within the aqueous matrix and preventing phase separation during storage and application. The resulting mixture formed a fine oil-in-water emulsion, with an estimated droplet diameter in the range of 1–10 µm, a size class typical of emulsions stabilized by polysaccharide-based surfactants. This droplet size ensured homogeneous spray ability, improved leaf surface coverage, and prolonged retention on the plant canopy. The components were incorporated under continuous stirring to ensure complete emulsification and a consistent droplet size distribution suitable for foliar applications.

### 2.3. Chemical Analysis of SGE

Total phenolic content (TPC) of SGE was determined by Folin–Ciocalteu reagent; the measurement was conducted on a Cary 100 Conc UV-Vis spectrophotometer (Agilent, Santa Clara, CA, USA) at λ = 760 nm against blank. Gallic acid was used as standard phenolic compound to make the calibration curve that ranges between 0 and 500 mg L^−1^ (R^2^ = 0.992). The results were expressed as gallic acid equivalent (GAE) in g of dry weight. Total flavonoid content (TFC) was estimated using AlCl_3_ according to Rongai [21]. TFC was expressed as rutin equivalent (mg L^−1^) of the extract and a calibration curve was generated.

Total acidity was determined by titration method with a 0.01 N alkaline sodium hydroxide solution using phenolphthalein (1%) as indicator and was expressed in g L^−1^ citric acid. The pH value of SGE was determined with a Hamilton pH electrode sensor.

The HPLC-DAD-ESI/MS analysis was carried out according to Rongai [22]; the diode array detector performed simultaneous monitoring at 280nm and 320 nm. The mobile phase consisted of 2% (*v*/*v*) acetic acid in water (eluent A) and 2% (*v*/*v*) acetic acid in methanol (eluent B). The flow rate was constant at 0.9 m L min^−1^ split for the MSD with a split ratio of 7:3. The ESI interface operated in Negative (ES^−^) and MRM (Multiple Reaction Monitoring) mode, monitoring two *m*/*z* mass transactions for each component investigated.

### 2.4. Toxicity Bioassays

Form was tested in the laboratory at increasing concentrations of 0.125, 0.25, 0.50, 1 and 2%. Deionized water was used as a negative control, and an additional test was performed with 12 mg/L deltamethrin (Decis^®^ Jet, Bayer, Leverkusen, Germany) as a positive control.

The bioassays, based on a modified version of the dip test by [17], were performed to test the toxic effect against *S. titanus* adults. Forty individuals per treatment (10 individuals × 4 replicates) were immersed for 15 s into 250 mL of solution and immediately transferred into a Petri dish (9 cm in diameter) containing a broad bean leaf sprayed with the same solution. Petri dishes were then heated to 24–25 °C. The percentage of American grapevine leafhopper mortality was recorded 1 h after the treatment. The percentage of mortality was calculated as corrected mortality (%) = [(mortality in treatment % − mortality in control %)/(100 − mortality in control %)] × 100. The mean lethal doses LD25, LD50 and LD75 were calculated from the resulting regression equation of the percentage mortality rate observed one hour after the treatment. Four replicates were performed, and each test was repeated twice.

### 2.5. Field Experiments

The efficacy of Form was assessed under field conditions during the 2024 and 2025 growing seasons in two commercial vineyards located in the Abruzzo region (central Italy). The first trial, focusing on adult *S. titanus*, was conducted in Fossacesia (CH), Italy (42°15′ N; 14°29′ E). In the subsequent year, the formulation was evaluated against nymphs in a vineyard situated in Scafa (PE), Italy (42°26′ N; 13°99′ E). Both vineyards consisted of *Vitis vinifera* L. cv. Montepulciano, trained on a traditional trellis system, with vine spacing of 2.5 m × 2.5 m, a configuration commonly used in central Italy to facilitate mechanical and manual operations.

The experimental design includes two blocks, each containing two plots (treated and untreated). In each plot, which covers an area of approximately 500 m^2^, four traps were installed. Plot dimensions were selected to reduce edge effects and ensure homogeneous exposure to the vineyard’s microclimatic conditions. A single foliar application of Form at 1% concentration was performed on 8 August 2024 for the adult trial and on 5 June 2025 for the nymph trial. Treatments were applied using a farm-mounted atomizer calibrated to deliver 1100 L/ha, operating at 8 bar pressure. The spray was directed onto both sides of the grapevine canopy as well as onto the inter-row ground vegetation, ensuring full coverage of potential *S. titanus* habitats. No insecticides or other crop protection products targeting leafhoppers were applied during the entire field trial to avoid confounding effects.

In 2024, adult population dynamics were monitored from 1 to 16 August 2024 using yellow sticky traps (20 cm × 25 cm), a standard method for detecting *S. titanus* adults in European vineyards. The traps were positioned in the lower portion of the canopy at approximately 1.5 m above ground, corresponding to the typical flight height of emerging adults. Traps were replaced on the day of monitoring to maintain optimal adhesive performance and avoid saturation by debris or non-target arthropods. The number of adults captured was counted separately on both sides of the traps under laboratory conditions. Adult density per plot was calculated as the cumulative number of individuals per trap over the monitoring period.

In 2025, nymphal populations were monitored from 5 to 12 June 2025, a period corresponding to the peak abundance of 2nd–4th instar nymphs in central Italy. In each plot, 100 leaves were randomly collected from the median portion of the shoots, which is the area where nymphs are most located. Leaves were examined in the field and further inspected in the laboratory when necessary. The number of nymphs per leaf was recorded and used to calculate two epidemiological parameters: (i) incidence, expressed as the percentage of leaves on which at least one nymph was present; and (ii) severity, defined as the mean number of nymphs per leaf based on the total of 100 leaves examined. Meteorological parameters were monitored throughout the trial period.

### 2.6. Statistical Analysis

Mortality data were analyzed by ANOVA with means separation by Fisher’s protected LSD test at α = 0.05. The data were arcsine-transformed prior to analysis to correct for heterogeneity of variance. Probit analysis (EPA Probit Analysis Program version 1.5) was used to estimate the lethal dose for 25, 50 and 75% of the population (LC25, LC50 and LC75). SigmaPlot V10 (London, UK) and Sigma Stat V3.5 (London, UK) were used to obtain the graphs.

## 3. Results

### 3.1. Chemical Analysis of SGE

Chemical analyses showed that SGE had a high TPC (212.48 mg GAE/g DW) and TFC (45.30 mg RE/g DW). Total acidity was 0.480 meq NaOH g^−1^, and the pH was 5.94. SGE was also analyzed by HPLC-DAD-ESI/MS, which revealed a high content of sesquiterpenes.

### 3.2. Toxicity Bioassays

**Figure 1:**
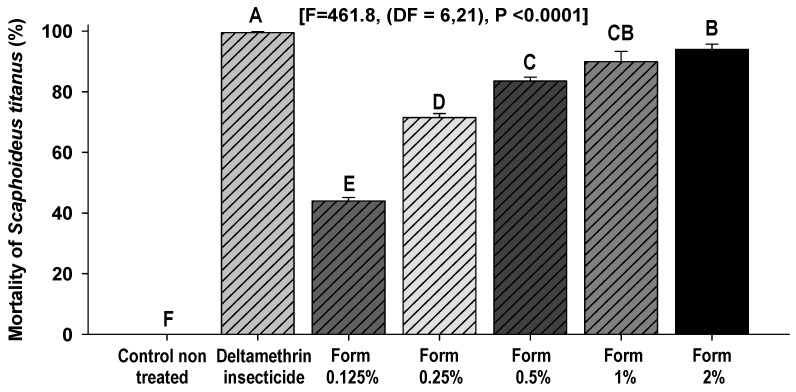
Mean percentage (±SD) of mortality of *S. titanus* adults recorded 1 h after treatment with different doses (0.125–2%) of Form and with deltamethrin. Values with different letters for each group are statistically different (LSD test, α = 0.05).

In the bioassays conducted with Form at a concentration of 0.5%, the mean mortality of *S. titanus* adults reached 84.0%, whereas at 2% it reached 94.3%. No significant differences were detected between the two highest concentrations of Form (1% and 2%) (Figure 1).

**Figure 2:**
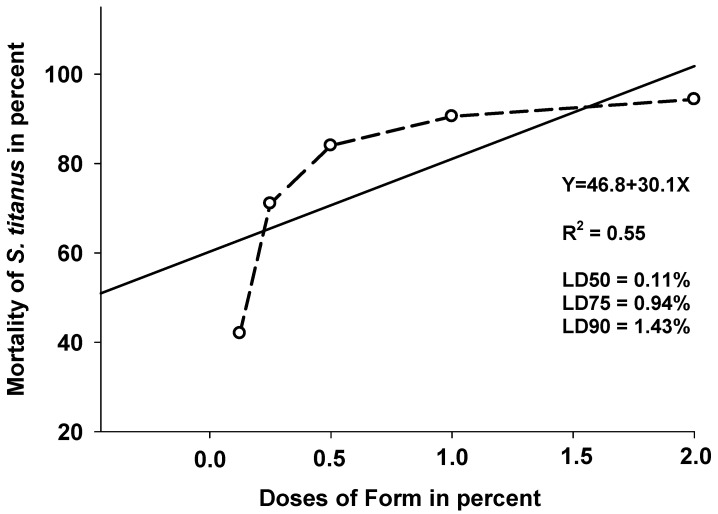
Lethal doses of different doses (0.125–2%) of Form on *S. titanus* 1 h after treatment.

This rate obtained with deltamethrin is according to [8] who reported that the mortality of *S. titanus* adults treated with synthetic insecticides (deltamethrin, lambda-cyhalothrin and sulfoxaflor) was 100% three days after treatment. Mortality was dose-dependent and increased with concentration. The corresponding lethal dose values were 0.11% (LD50), 0.94% (LD75) and 1.43% (LD90) (Figure 2).

### 3.3. Field Experiments

**Figure 3:**
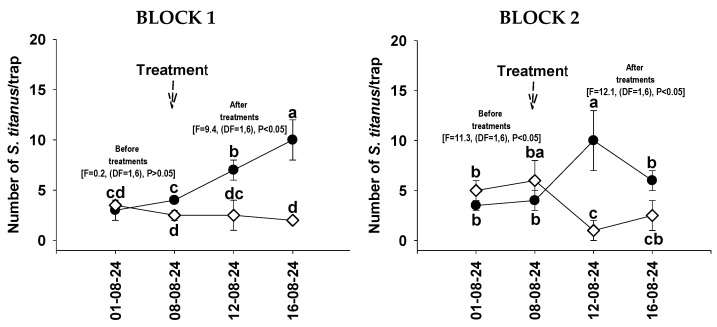
Number (±SD) of *S. titanus* adults captured from 1 August 2024 to 16 August 2024 in the two experimental blocks, after one treatment with Form at 1% concentration. Untreated plots ●-●; treated plots □-□. Values with different letters for each group are statistically different (LSD test, α = 0.05). Standard deviations of the means are indicated.

Before treatment (1 and 8 August 2024), the average number of *S. titanus* adults captured in the vineyard was 3.9/trap in both blocks. After treatment (12 August 2024), the average decreased significantly to 2/trap in the treated vineyard and increased to 8.2/trap in the untreated vineyard. Eight days after treatment (16 August 2024), the average capture rate in the treated vineyards remained low (2.4/trap), while in the untreated vineyard it was 8.0/trap (Figure 3). During the trial period in 2024, the maximum temperature (Tmax) ranged from 30.2 °C to 39.4 °C, while the minimum temperature (Tmin) ranged from 19 °C to 24 °C. Relative humidity (RH) varied between 45.2% and 65.1%, and no rainfall (0 mm) was recorded.

**Figure 4:**
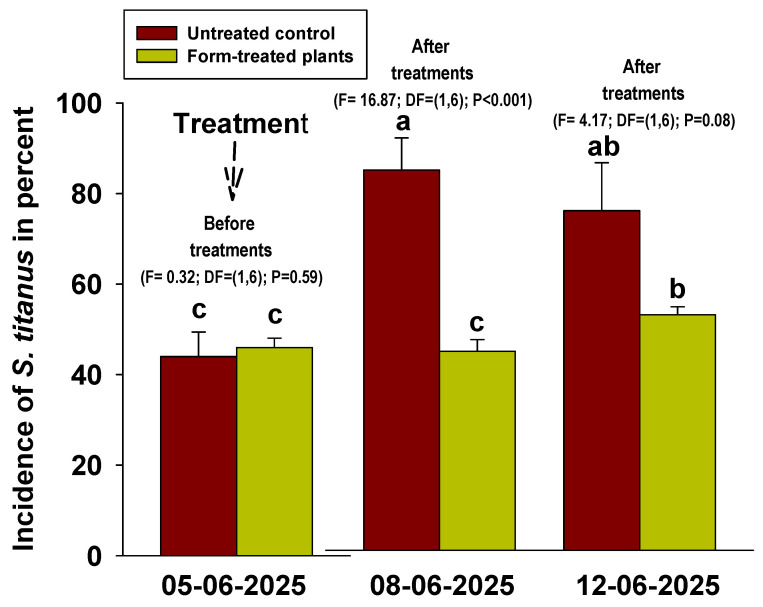
Percentage of leaves on which at least one nymph was present out of the 100 leaves examined. Values with different letters within each group are statistically different (LSD test, α = 0.05). Standard deviations of the means are shown.

Regarding nymph monitoring, before treatment, the incidence of *S. titanus* was 44.0% and 46.0% in the untreated and Form-treated plants, respectively. After treatment (8 June 2025), the percentage of leaves with *S. titanus* nymphs increased to 84.0% in the untreated vineyard, while it remained stable at 44.0% in the Form-treated area. A similar trend was recorded on 12 June 2025 (Figure 4). During the trial period in 2025, the maximum temperature (Tmax) ranged from 29.8 to 33.7 °C, while the minimum temperature (Tmin) ranged from 12.3 to 15.6 °C. Relative humidity (RH) varied from 71.8% to 79.2%, and total rainfall was 25 mm.

Monitoring was also conducted to assess the severity of the *S. titanus* infestation. Before treatment, severity was 0.24 and 0.26 nymphs per leaf in the untreated and Form-treated plants, respectively. On 8 June 2025, three days after treatment, the average number of *S. titanus* nymphs per leaf increased to 0.76 in the untreated vineyard, whereas it was 0.43 nymphs per leaf in the Form-treated area. On 12 June 2025, seven days after treatment, the average number of *S. titanus* nymphs per leaf remained lower in the treated vineyard than in the untreated one; however, the difference between the two values was not statistically significant (Table 1).

## 4. Discussion

Our results demonstrate that Form exerts a measurable toxic effect on *S. titanus* adults under both laboratory and field conditions. In laboratory bioassays, concentrations of 0.5–1% induced substantial mortality, although their efficacy remained lower than that of deltamethrin. This outcome is consistent with the generally lower potency expected from botanical formulations compared with synthetic pyrethroids.

Comparison with the findings of Prazaru [8] further contextualizes these results. Their study demonstrated that the residual deposits of deltamethrin, lambda-cyhalothrin, and sulfoxaflor remained fully active three days after application, maintaining 100% mortality. Importantly, their assessment focused exclusively on residual efficacy rather than on topical activity. Such high residual persistence is characteristic of these synthetic insecticides, which are designed to remain stable on treated surfaces for several days or even weeks. In contrast, natural formulations such as Form typically degrade more rapidly due to their volatile or biodegradable components. This difference partially explains the disparity in absolute mortality values between synthetic standards and Form.

Despite its lower intrinsic potency and expected reduced persistence, the toxic action of Form observed in controlled conditions was effectively translated into a measurable effect in the field. In the treated vineyard, adult *S. titanus* captures declined significantly following application, indicating that Form can suppress adult populations under realistic vineyard conditions. This finding is particularly relevant because adult stages play a key role in pathogen dissemination, especially in the case of *flavescence dorée*. Reducing adult abundance even without achieving complete control may contribute meaningfully to vector management and disease mitigation.

In one of the monitored vineyards, approximately 1100 L ha^−1^ of spray mixture was applied. Although this application volume is higher than those typically required for conventional insecticides, it suggests that Form-based treatments may represent a viable component of integrated pest management strategies, particularly where reducing synthetic pesticide inputs is a priority.

A possible explanation is the occurrence of a synergistic effect between the plant-derived constituents. As reported by Rongai [17], the combination of vegetal oil, *C. annuum* ‘*Cayenne*’, and *S. guaranitica* exhibits strong toxic activity against *P. spumarius*, exceeding the effects of each component applied individually.

One of the most remarkable and unexpected findings of this study is that Form exhibited greater efficacy against adults of *S. titanus* than against nymphs. In the nymph trial, carried out in 2025, no significant differences were detected between treated and control plots seven days after the application, indicating an overall limited impact on immature stages. This result contrasts sharply with the general pattern observed for the majority of insecticidal compounds, to which nymphs are typically more susceptible than adults due to their thinner cuticle, lower detoxification capacity, and reduced mobility.

The observed discrepancy in efficacy may be attributable to several biological and ecological factors. A first hypothesis is that Form may possess a strong repellent or irritant effect on adults, prompting them to move away from the treated canopy shortly after exposure. Such behavioral responses are well documented for essential oils, plant extracts, and bioactive natural emulsions, many of which can alter insect orientation, feeding behavior, or host-plant selection. In the present study, adults may have emigrated from treated plots toward untreated areas, thereby producing an apparent reduction in population density in the treated vineyard. Because yellow sticky traps were placed within each plot, the spatial redistribution of adults could have amplified the measured differences between treatments.

Another possible explanation relates to the feeding habits and mobility of different developmental stages. Adult *S. titanus* are highly mobile, capable of sustained flight and rapid movement between adjacent vines. Nymphs, on the other hand, are mostly sedentary, confined to the abaxial leaf surface, and tend to remain protected within the canopy [12,23]. The deposition of Form on the underside of leaves—where nymphs primarily reside—may have been lower than on the outer canopy surfaces, thus limiting direct exposure during immature stages. In contrast, adults actively landing on or walking across treated surfaces may have been more effectively exposed to the formulation.

Physiological factors may also play a role. The bioactive components of Form, derived from *S. guaranitica* extract and capsicum-infused oil, could affect adult sensory or neurological pathways more strongly than those of nymphs. Terpenoids, flavonoids, and phenolic compounds present in the phytochemical profile of the genus Salvia are known for their bioactivity against insects [24,25]. Capsaicinoids and other secondary metabolites are known to modulate insect chemoreception, often resulting in deterrent or repellent effects in several Hemiptera [26,27]. These compounds can interfere with host selection and feeding behavior by altering sensory perception and plant acceptability. It is therefore plausible that, if adult insects are more responsive to these compounds than immature stages, their avoidance behavior may have contributed to the significant population decline observed in treated plots.

Overall, the contrasting efficacy observed between adults and nymphs suggests that Form may not act as a conventional toxicant but may instead exert a multifaceted action involving repellence, irritation, or deterrence. These characteristics, while not aligned with classical insecticidal modes of action, could nevertheless be valuable within integrated pest management (IPM) programs. Specifically, adult repellence early in the season could reduce the number of infectious individuals entering vineyards, thereby lowering the risk of *flavescence dorée* transmission. Further studies, particularly behavioral assays, residue distribution analyses, and dose response bioassays, are essential to clarify the underlying mechanisms and to optimize application strategies.

Plant oils, including olive oil, are generally considered harmful to insects because they obstruct the spiracles and impair respiration. However, despite this plausible physical mode of action, several studies have shown that oils used alone often result in low or negligible mortality [16]. This discrepancy suggests that spiracle blockage alone may not be sufficient to induce substantial mortality in many Hemipteran species. For instance, oil-based treatments applied against juveniles of *Philaenus spumarius* and *Neophilaenus campestris* produced very low mortality rates [28]. Similarly, vegetable oil applied against the California red scale (*Aonidiella aurantia*) resulted in only moderate mortality (approximately 46%) [18]. Other authors [10] also reported that orange oil is ineffective against *S. titanus*. Collectively, these findings indicate that plant oils alone rarely achieve reliable field control, underscoring the importance of combining them with other bioactive plant extracts to enhance efficacy.

Among these extracts, *S. guaranitica* contains high levels of cirsiliol and caffeic acid ethyl ester, both of which are documented to possess insecticidal activity. Abdelshafeek [29] reported that flavonoids such as cirsiliol, luteolin, and chrysoeriol exhibit significant bioactivity against *Phloeotribus oleae*. Furthermore, some authors [30] demonstrated that caffeic acid exerts toxic effects on *Helicoverpa armigera* Hübner by inhibiting key intestinal proteases, thereby impairing larval digestion and development.

The third plant-derived component of Form, *C. annuum*, also contributes to its overall activity. Okonkwo [13] attributed the insecticidal properties of *C. annuum* to the alkaloids, flavonoids, anthocyanins, tannins, and saponins present in its fruits, while Boulogne [14] highlighted the roles of eugenol and pulegone. However, when tested individually, *C. annuum* extracts have consistently shown weak insecticidal activity [19,20], reinforcing the hypothesis that the efficacy of Form arises from synergistic interactions among its components rather than from any single extract.

Overall, the results of this study indicate that a plant-origin formulation such as Form represents a promising alternative to chemical insecticides for controlling the main vector of grapevine *flavescence dorée* phytoplasma. Its integration into sustainable pest management programs could contribute to reducing reliance on synthetic insecticides while maintaining effective control of *S. titanus* populations.

## Figures and Tables

**Table 1:** Average number of *S. titanus* nymphs per leaf, based on samples of 100 grapevine leaves collected from 5 June 2025 to 12 June 2025. Values with different letters are statistically different (LSD test, α = 0.05). Standard deviations of the means are shown.

Groups	Doses	Sampling Dates		
		5 June 2025	8 June 2025	12 June 2025
Untreated control		0.24 ± 0.06 d	0.76 ± 0.11 a	0.66 ± 0.2 ab
Treated	1%	0.26 ± 0.04 d	0.43 ± 0.02 c	0.62 ± 0.01 b
		F = 0.32 dF = (1, 6); *p* = 0.59	F=23.3 dF = (1, 6); *p* = 0.0023	F = 0.12 dF = (1, 6); *p* = 0.74

## Data Availability

The original contributions presented in this study are included in the article. Further inquiries can be directed to the corresponding author.
